# Base on balls for the Chapman strategy: Reassessing Brouwer, Brenner, and Smeets (2002)

**DOI:** 10.3758/s13414-012-0328-6

**Published:** 2012-06-22

**Authors:** Frank T. J. M. Zaal, Raoul M. Bongers, Gert-Jan Pepping, Reinoud J. Bootsma

**Affiliations:** 1 Center for Human Movement Sciences, University Medical Center Groningen, Sector F, University of Groningen, P.O. Box 196, NL-9700 AD Groningen, The Netherlands; 2 Institut des Sciences du Mouvement E. J. Marey, Aix-Marseille Université, Marseille, France

**Keywords:** Perception and action, Locomotion, Motion perception

## Abstract

A true understanding of skilled behavior includes the identification of the information that underlies the perception–action cycle at work. Often, observers’ sensitivity to perceptual variables is established in laboratory-situated simulation-based psychophysical experiments. The observers’ sensitivity thus determined is then used to draw conclusions that will generalize the findings to natural behavior. Focusing on the example of running to catch fly balls, the present contribution takes the study of Brouwer, Brenner, and Smeets (Perception & Psychophysics 64:1160–1168, 2002) to illustrate how common assumptions in the steps from psychophysical experiments to natural behavior can result in ungrounded conclusions. These authors built an argument to reject the use of the Chapman strategy of zeroing out optical acceleration. For this argument, they determined the sensitivity of the visual system to acceleration, assuming that acceleration is detected as a velocity ratio. Next, they showed that catchers started running earlier than could be expected on the basis of sensitivity thresholds for this velocity ratio, concluding that running initiation could not have been based on optical acceleration. In the present study, we argue that important assumptions in the Brouwer et al. (Perception & Psychophysics 64:1160–1168, 2002) line of argument are incorrect. First, we show how the assumption of parabolic ball flight trajectories, although convenient, biased Brouwer et al.’s (Perception & Psychophysics 64:1160–1168, 2002) conclusion. Next, we present an experiment revealing that observers do not base their judgments of acceleration on the velocity ratio. Thus, we demonstrate that Brouwer et al.’s (Perception & Psychophysics 64:1160–1168, 2002) argument that optical acceleration cannot serve as the information for running to catch fly balls does not hold.

There is a widespread belief that the hardest ball for an outfielder to catch is one that comes straight at her/him (e.g., Adair, 2002). This kind of ball has intrigued not only spectators and players of ball games, but likewise scientists trying to unravel the perception–action cycle that underlies the successful interception of baseballs, cricket balls, soccer balls, Frisbees, and the like (e.g., Adair, 2002; Babler & Dannemiller, 1993; Bongers & Michaels, 2008; Brouwer, Brenner, & Smeets, 2002; Fink, Foo, & Warren, 2009; McBeath, Shaffer, & Kaiser, 1995; McLeod & Dienes, 1996; McLeod, Reed, & Dienes, 2001, 2003, 2006; McLeod, Reed, Gilson, & Glennerster, 2008; Michaels & Oudejans, 1992; Oudejans, Michaels, & Bakker, 1997; Shaffer, Krauchunas, Eddy, & McBeath, 2004; Shaffer & McBeath, 2002; Shaffer, McBeath, Krauchunas, & Sugar, 2008; Todd, 1981; Zaal & Michaels, 2003). For such balls flying along the sagittal plane, a strategy has been formulated that will bring the catcher-to-be to the interception point right at the moment that the ball will arrive there, without actually having to know the interception location or interception time. That is to say, rather than having to be able to perceptually establish the ball trajectory and infer the landing position and time of arrival of the ball (cf. Adair, 2002; Brancazio, 1985; Chodosh, Lifsin, & Tabin, 1995; Saxberg, 1987a, 1987b), information exists that tells the catcher-to-be whether or not the current running speed will bring her/him to the interception location right on time to make the catch. The strategy has come to be known as the *Chapman strategy* or the *strategy of optical acceleration cancelation*.

In 1968, the physicist Seville Chapman (see also Michaels & Oudejans, 1992; Todd, 1981) demonstrated that, for a baseball traveling with a parabolic trajectory along the sagittal plane to a fielder running at constant speed to the interception location, in such a way that he or she will arrive at the landing location of the ball at the moment that the ball lands there, the rate of change of the tangent of the elevation angle *α*—the angle between the horizontal and the line connecting the ball and the fielder’s point of observation—remains constant. Should the fielder not arrive at the landing site of the ball in time, then the rate of change of tan(*α*) will not be constant: An increasing rate of change will instead occur when the ball will fly over the head of the fielder, or a decreasing rate of change will occur when the ball will cross at eye level in front of the fielder’s head.^1^ In these situations, to catch the ball, the fielder should change running speed in such a way that the rate of change of tan(*α*) becomes constant again. For instance, when running backward, an increasing rate of change of tan(*α*) tells the fielder to increase running speed, until the rate of change of tan(*α*) becomes constant; continued running at the speed at which optical acceleration is zero will eventually lead to getting to the interception location right at the time that the ball will arrive there. Also, when running forward, a decreasing rate of change informs the fielder that he or she should increase the forward running speed until the rate of change of tan(*α*) is constant, and this running speed will get him or her to the interception location in time.

The Chapman strategy is also known as the strategy of optical acceleration cancelation (e.g., Fink et al., 2009; Kistemaker, Faber, & Beek, 2009; McLeod et al., 2006; Michaels & Oudejans, 1992; Zaal & Michaels, 2003) because the rate of change of tan(*α*) is equivalent to the speed of the projection of the ball along an image plane (see, e.g., Brouwer et al., 2002; Michaels & Oudejans, 1992; Zaal & Michaels, 2003). A positive rate of change of tan(*α*) amounts to a positive rate of change of optical speed—that is, optical acceleration—and a negative rate of change of tan(*α*) amounts to a negative rate of change of optical speed, or optical deceleration. The fielder’s task, thus, is to get rid of any optical acceleration,^2^ positive or negative, and he or she will arrive at the interception point right on time to make a catch.

Previous studies have shown that participants make judgments and run in ways consistent with the Chapman strategy. For instance, Zaal and Michaels (2003) had participants judge whether approaching virtual balls would cross eye level behind or in front of their heads, and found support for the use of optical acceleration in the patterns of response times. Also, when looking at running patterns, a number of studies have demonstrated that catchers adapt their running speeds in such a way that optical acceleration remains close to zero (e.g., McLeod & Dienes, 1996; McLeod et al., 2001; Michaels & Oudejans, 1992; Zaal & Michaels, 2003). Furthermore, the use of the Chapman strategy implies that the interception location is not known or calculated beforehand—that is, before the catcher starts running (i.e., the information is prospective rather than predictive). Thus, depending on how a ball arrives at some interception location (flying higher or lower paths, taking shorter or longer times for its flight), catchers are expected to run to that interception location with different running patterns. This was confirmed by McLeod and colleagues, in a study on the catching of real cricket balls (McLeod & Dienes, 1996) and in a study on the heading of virtual soccer balls in a virtual-reality experiment (McLeod et al., 2008; see also Fink et al., 2009). In sum, participants in a number of studies have behaved in ways consistent with the use of optical acceleration.

Despite this evidence in favor of the Chapman strategy, Brouwer et al. (2002) concluded that the running behavior of fly-ball catchers could not be based on the information provided by optical acceleration. More precisely, they claimed that the sensitivity to optical acceleration was simply not good enough to even know in which direction to start running at the time that fly-ball catchers actually started their movement to the interception location. To arrive at this conclusion, Brouwer et al. (2002) performed psychophysical experiments to establish a sensitivity threshold for acceleration and subsequently used data from a real fly-ball-catching experiment (Oudejans et al.’s, 1997, study) to compare running-initiation times (indicating the times at which catchers had apparently made an informed decision to start running forward or backward) with the times that were needed to reach that sensitivity threshold of optical acceleration (see Fig. 1A). Brouwer et al. (2002) found that the decision to start running occurred earlier than the moment that optical acceleration had reached the sensitivity threshold. On the basis of this finding, they concluded that the decision to start running in the right direction could not have been based on optical acceleration as information. If the analysis presented by Brouwer et al. (2002) were to be correct, the Chapman strategy would remain an interesting example of how the perception–action loop might work, but the bottom line would be that things go differently in the real world of running to catch a fly ball.

**Fig. 1:**
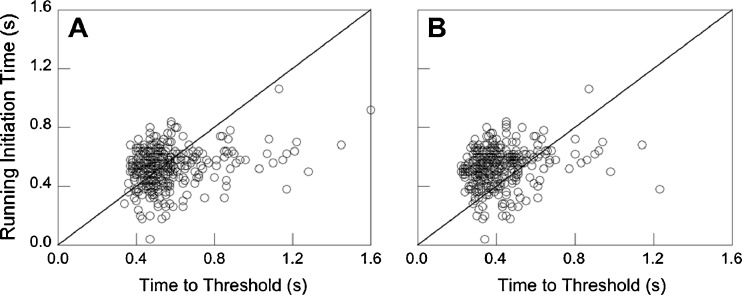
Running-initiation times from the Oudejans et al. (1997) study as a function of reaching a velocity-ratio threshold of 25 %, assuming parabolic ball trajectories (A) or assuming air resistance (B). See the text for details. Panel A replicates the data from Fig. 6 in Brouwer, Brenner, and Smeets (2002)

In the present contribution, we carefully examined the line of argument developed by Brouwer et al. (2002). To anticipate, we will conclude that their (dismissive) conclusion regarding the Chapman strategy was, at best, premature. More specifically, we address the effects of two important assumptions made by Brouwer, Brenner, and Smeets: (1) the assumption that ball flight trajectories can be approximated by parabolic curves and (2) the assumption that observers respond to the velocity ratio when asked to judge acceleration. Both are important assumptions featuring in their computations of the times to reach the proper acceleration sensitivity thresholds.

## The assumption of parabolic flight trajectories

Ideally, when aiming at understanding natural behavior, one would like to study a task that most closely resembles that natural behavior. When hoping to find out whether and how people use optical acceleration to start and guide their running to catch fly balls, the best setting would be one in which participants have to catch real balls. One of the disadvantages of such a natural setting (see Zaal & Bootsma, 2011), however, is that one must be able to accurately determine the displacement of the ball and the participant. This has proven to be quite a challenge. In particular, accurately tracking free-flying balls is both difficult and time-consuming. An alternative would be to reconstruct ball trajectories on the basis of a small set of measured key ball positions. For instance, McLeod and colleagues (McLeod & Dienes, 1996; McLeod et al., 2001, 2006) estimated ball trajectories from measured distances, flight times, ball launching angles, and speeds, taking into account air resistance as a force related to the square of ball speed (cf. Brancazio, 1985). Bongers and Michaels (2008) used a set of the first few ball positions and positions around the zenith to extrapolate the ball trajectory from a model that they had developed from a series of full ball trajectories. The model included second- and third-order polynomials of horizontal and vertical positions, respectively, as a function of time. Importantly, both of these procedures acknowledged the fact that ball trajectories are not parabolically shaped, because of the drag that balls experience when flying through the atmosphere. In contrast, however, Brouwer et al. (2002) assumed that the effects of air resistance could be ignored, in that their analyses included the assumption that ball trajectories *could* be approximated by parabolas.

To compute the times to reach the optical-acceleration threshold, Brouwer et al. (2002) used data provided by the authors of the Oudejans et al. (1997) study. These data included, for each trial, the duration of the ball flight and the distance that the ball had traveled. From these flight durations and distances, ball flight trajectories were reconstructed. When the flight path of a ball is parabolic, its horizontal and vertical positions (*x* and *z*, respectively) are well-known functions of time *t*:

$$ x = {p_x}_0 + {v_x}_0t, $$

$$ z = {{p}_{z}}_{0} + {{v}_{z}}_{0}t - {{1} \left/ {2} \right.}\,g\,{{t}^{2}}, $$

in which *p*_*x*0_ and *p*_*z*0_ are the ball’s initial horizontal and vertical positions, respectively, relative to the point of observation; *v*_*x*0_ and *v*_*z*0_ are the ball’s initial horizontal and vertical velocities, respectively; and *g* is the acceleration due to gravity (9.81 m/s^2^). Flight duration *T* and distance traveled *D* determine the initial horizontal and vertical velocities:

$$ {v_x}_0 = D/T, $$

$$ {{v}_{{z0}}} = {{1} \left/ {2} \right.}\,g\,t. $$

**Fig. 2:**
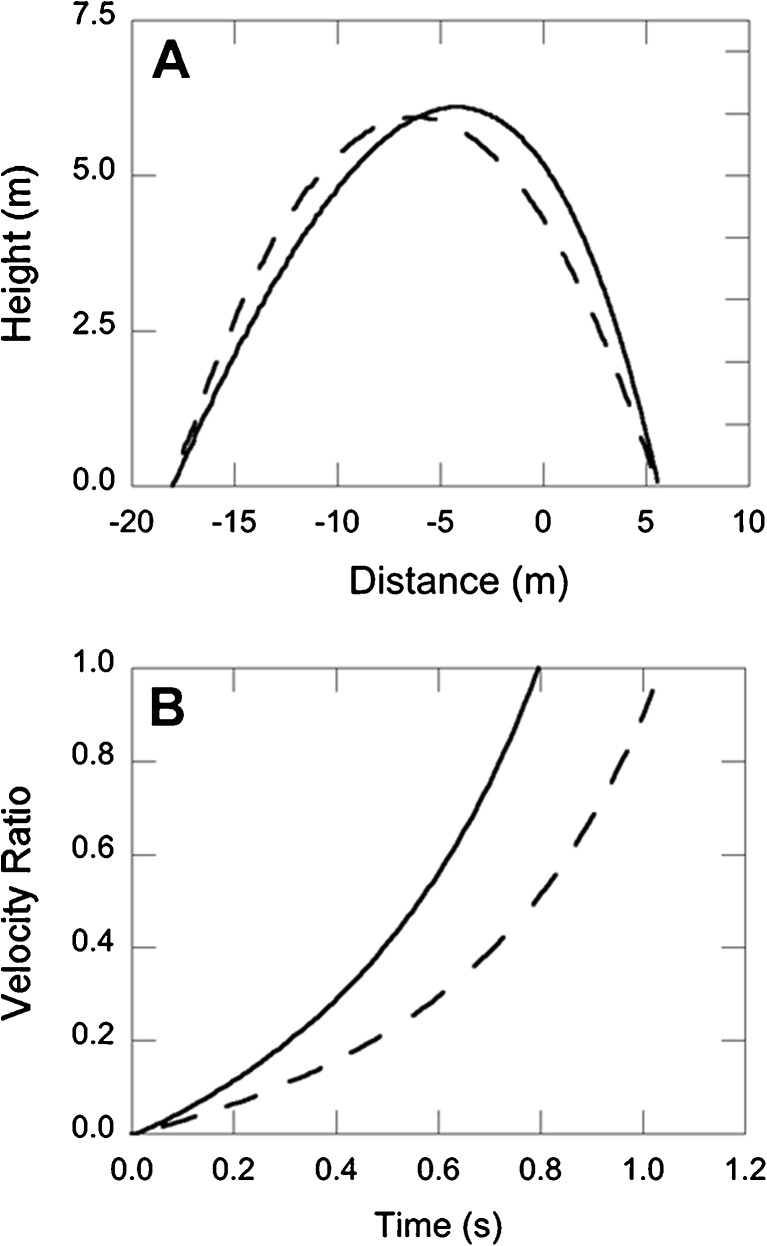
Example of a parabolic ball trajectory (dashed lines) and a ball trajectory with air resistance (solid lines), both with the same distance *D* and flight duration *T*. Panel A shows the ball trajectories, and panel B shows the velocity-ratio values computed from the ball trajectories. In this example, the ball was launched from 18.0 m and passed eye level 5.58 m behind the stationary participant. Ball flight time was 2.20 s

As a result, the reconstruction of the horizontal and vertical ball positions as a function of time can be performed analytically. Figure 2A (dashed line) gives an example of a parabolic ball trajectory determined in this way from a given ball flight time and distance (taken from the Oudejans et al., 1997, data set).

In a next step, optical variables can be computed by determining the projection of the reconstructed physical ball trajectory onto an image plane. Optical position equals the ratio of a ball’s vertical and horizontal positions (*z* and *x*), and optical velocity is the derivative of position with respect to time. Brouwer et al. (2002) assumed that optical acceleration is detected as a velocity ratio (we will return to this assumption later): The velocity ratio at some time *t* is defined as the change in optical velocity from *t*_0_ to *t*, divided by the average optical velocity over the interval from *t*_0_ to *t*. The dashed line in Fig. 2B shows the velocity ratio as a function of time for the corresponding parabolic flight path of Fig. 2A.

In a final step, for each and every trial, the time at which the velocity-ratio time series reaches a certain velocity-ratio threshold can be determined. The threshold used by Brouwer et al. (2002) was 0.25, on the basis of their experiments in which participants judged the acceleration of a horizontally moving dot on a computer screen. Figure 1A gives the actual running-initiation times (taken directly from the Oudejans et al., 1997, data) as a function of the threshold times computed by Brouwer et al. (2002). Figure 1A (a replication of Fig. 6 in Brouwer et al.’s study) shows that only 48 % of the data points fall above the identity line, which would imply that in roughly half of the trials the time to reach the threshold of optical acceleration (i.e., a velocity ratio of 0.25) preceded the moment that the participants had started running.^3^

In reality, all balls that fly through the atmosphere experience air resistance. As a result, ball trajectories deviate from the parabolic trajectory along which they would fly in a vacuum. For instance, baseballs fly about half the distance that they would without air drag (cf. Adair, 2002). To assess the effects of air resistance on the ball trajectories and their associated optics, we used numerical integration to solve the equation of motion of a projectile with drag. The combined forces on a ball (which determine its acceleration *a*) are a sum of the gravitational force *F*_*g*_ and the drag force *F*_*d*_ (which is a function of squared velocity *v*):

$$ ma = {F_g} + {F_d}, $$

$$ {F_g} = - mg, $$

$$ {F_d} = - {c_d}\rho A{v^2}, $$

with a mass of the ball *m*, air density *ρ*, and projected ball area *A* (which equals *π* times ball radius *r* squared). For each trial of the Oudejans et al. (1997) data set, we used a standard MATLAB minimization procedure to find the trajectory that would cover the distance *D* in the time *T* as specified for that specific trial. For the simulations, we estimated the ball characteristics as well as the atmospheric circumstances at the time of the Oudejans et al. experiment. Tennis balls come in many varieties but have to conform to standard regulations. Furthermore, we know that the experiments took place in a sports hall in Amsterdam. With this information, we used the following values for the parameters in our simulations: *m* = 58 g, *r* = 3.25 cm, *ρ* = 1.225 kg/m^3^ (at 15 °C at sea level), and *c*_*d*_ = 0.6 (cf. Goodwill, Chin, & Haake, 2004). The solid line in Fig. 2A presents an example of a ball trajectory determined in this way. The trajectory is clearly not parabolic for this trial. More importantly, the optical-velocity time series, given in Fig. 2B, differs significantly from the time series for the parabolic ball trajectory. If we take into account air resistance, a certain threshold of the velocity ratio is reached earlier (about 180 ms for the velocity-ratio threshold of 0.25 in the example of Fig. 2) than when air resistance is assumed to be nonexistent. As a consequence, the Brouwer et al. (2002) plot of actual running-initiation times as a function of velocity-ratio threshold times would look different. That is, it would lead to a cloud of points more to the left, and in the majority of cases above the identity line (Fig. 1B): We determined that when ball trajectories are computed with air drag, 78 % of the data points fall above the identity line.

One could argue that a number of 78 % of the trials above the identity line is certainly better than the 48 % used to conclude against the Chapman strategy, but that in a fair number of trials participants apparently still started running before they could have detected optical acceleration. At least three remarks can be made here. First, a closer inspection of the data points below the identity line shows that the points to the right of the main cloud of points (i.e., points with threshold times larger than 0.75 s) are all from trials in which the ball ended up very close (within 1.5 m) to the initial position of the catcher. Furthermore, in all but one of these trials, the ball crossed eye level behind the participants’ heads. Note that the Chapman strategy is a running strategy, to bring the catcher within a reachable distance from the approaching ball. The running is followed by the actual catching, about which the Chapman strategy has nothing to say. Balls crossing eye level about 1.5 m behind a catcher can easily be caught by stretching the arm combined with some body movement. This means that the movement belonging to the points with large threshold times probably was not related to running to catch (the Chapman strategy), but more to the subsequent catching itself. If so, these points should not be considered, and might be removed from the graph. Second, it might be the case that the threshold value of 0.25 proposed by Brouwer et al. (2002) is too conservative. Brouwer et al. (2002) determined these thresholds for stimuli of short durations, from the argument that catchers typically start running within 500 ms after ball launch (see also Brouwer, López-Moliner, Brenner, & Smeets, 2006). For this reason, they determined thresholds for stimuli of short duration. Babler and Dannemiller (1993) had previously considered stimuli of longer durations and had arrived at a threshold value of 0.20. In fact, Brouwer et al.’s (2002) data even suggest that the thresholds are lower at longer stimulus durations. The effect of employing a lower threshold is that the percentage of points above the identity line increases. A threshold value of 0.20 results in a total of 88 % of the points above the identity line. Establishing that acceleration detection thresholds would be lower than the 0.25 or 0.20 suggested by Brouwer et al. (2002) and Babler and Dannemiller, respectively, could be a goal for subsequent research. Finally, yet another possibility is that not only the threshold per se, but also the variable for which this threshold was determined (i.e., the velocity ratio) is problematic. This third possibility will be explored in the next section.

## The assumption of sensitivity to the velocity ratio

As mentioned, Brouwer et al. (2002) started their study by determining the sensitivity for optical acceleration. Their analyses on the times to start running assumed that observers used a velocity ratio (a variable that they called *change in velocity*) to detect optical acceleration. The experiment that we will present indicates that observers do not use this variable when judging acceleration.

The sensitivity of the visual system to acceleration has been tested in numerous studies (e.g., Babler & Dannemiller, 1993; Brouwer et al., 2002; Calderone & Kaiser, 1989; Filion, 1964; Gottsdanker, Frick, & Lockard, 1961; Haarmeier & Thier, 2006; Hick, 1950; Notterman, Filion, & Mandriota, 1971; Notterman & Page, 1957; Schmerler, 1976; Watamaniuk & Heinen, 2003; Werkhoven, Snippe, & Toet, 1992). An important observation/finding in these studies has been that, when asked to judge the acceleration of moving stimuli,^4^ observers do not respond to acceleration as physics would define it (the instantaneous rate of change of velocity *v*: *dv*/*dt*), even when the velocity of the stimulus changes at a constant rate (i.e., when *dv*/*dt* is constant). If observers based their judgments on *dv*/*dt*, these judgments should not be affected by other factors. Yet the sensitivity thresholds determined for *dv*/*dt* do vary as a function of stimulus presentation duration and average velocity (Babler & Dannemiller, 1993; Brouwer et al., 2002; Gottsdanker et al., 1961). Apparently, acceleration judgments are based on something other than *dv/dt*.

Babler and Dannemiller (1993) examined the entire psychophysical space rather than only focusing on the sensitivity thresholds derived from the psychometric functions. Babler and Dannemiller presented observers with stimuli that contained either acceleration or deceleration. Apart from varying the rates of acceleration across their stimuli, they also varied the average velocity of their stimuli. The observers’ task was to indicate whether they had seen acceleration or deceleration in the animation that they had been watching. Next, Babler and Dannemiller plotted the percentages of acceleration judgments as a function of *dv*/*dt* (much like Fig. 4, but with *dv*/*dt* on the abscissa instead of the velocity ratio). They observed that the psychometric curves for the different average velocities were clearly different from each other, with higher average velocities having curves more to the left (i.e., relatively more acceleration judgments). This would not have been the case if *dv*/*dt* had captured the unique variable that the observers were responding to. If the visual system detects acceleration on the basis of *dv*/*dt*, the fact that stimuli differ in, for instance, their average velocity should not affect the responses. Interestingly, the curves did overlap when they were plotted not as a function of *dv*/*dt*, but rather as a function of a variable that Babler and Dannemiller called the velocity ratio (*vr*): the change in (optical) velocity over the stimulus interval divided by the average (optical) velocity of the stimulus.^5^ The effect of variations in average velocity on the psychometric curves seen when the percentages of acceleration judgments were plotted as a function of *dv*/*dt* disappeared when the same percentages were plotted as a function of *vr*. With the overlapping curves obtained by plotting the psychometric curves as a function of the velocity ratio, Babler and Dannemiller seemingly had identified the variable that human observers actually respond to when asked to judge the presence of acceleration. Subsequently, many studies have adopted this variable to make inferences about the use of optical acceleration in such tasks as reaching and catching (e.g., Benguigui, Ripoll, & Broderick, 2003; Dubrowski & Carnahan, 2002; Lee, Port, & Georgopoulos, 1997; Port, Lee, Dassonville, & Georgopoulos, 1997; and also Brouwer et al., 2002, who labeled this variable *change in velocity*). Curiously enough, however, the Babler and Dannemiller study—the very study that seemed to have established velocity ratio as the perceptual variable used to judge acceleration—also provided a first hint that this variable does not fully fit the bill. Whereas in their first two experiments they designed their stimuli such that acceleration (*dv*/*dt*) was an independent variable, in their third experiment they used the velocity ratio as the independent variable. Having varied both the levels of the velocity ratio and the average velocity of their stimuli, Babler and Dannemiller reported, in passing, an effect of average velocity on their psychometric curves: As had been true for the *dv*/*dt* curves earlier, the curves of the percentages of acceleration judgments plotted as a function of *vr* were different for the different average velocities of the stimuli. This is not what should happen if the velocity ratio were the variable used for detecting acceleration. Furthermore, the detection thresholds for velocity ratios, computed from those curves, significantly differed across different average velocities. Taken together, these findings suggest that the velocity ratio might not be the unique perceptual variable that is used to detect optical acceleration.

## Experiment

The experiment reported here aimed at replicating the effect of average velocity on the judgment of acceleration and deceleration (i.e., an increase or decrease, respectively, in optical velocity) as reported by Babler and Dannemiller (1993). To allow for more direct comparisons with Brouwer et al.’s (2002) study, our experiment differs in a number of respects from Babler and Dannemiller’s Experiment 3. First, we asked our observers to judge acceleration and deceleration (as in Babler and Dannemiller’s, 1993, Exps. 1 and 2) rather than to judge whether the simulated fly ball would cross eye level behind or in front of the point of observation (as in Babler & Dannemiller’s, 1993, Exp. 3). Second, although we appreciate that the optics of approaching fly balls do not follow linear velocity changes (see Fig. 2B and note 3; cf. Babler & Dannemiller, 1993; Zaal & Michaels, 2003), we did use stimuli with constant (linear) changes in velocity. Our primary goal was to evaluate the effect of average velocity on acceleration judgments. A secondary goal was to determine whether such average-velocity effects were indeed due to differences in average velocity and not to differences in the constituent variables (i.e., duration and amplitude). In the situation of uniform velocity changes, average velocity can be computed by dividing the movement amplitude by the duration of the stimulus. This implies that average velocity can be varied by varying movement amplitude and/or by varying movement duration. To parse out the effects of average velocity, movement amplitude, and movement duration, we used a design with three groups of participants (a constant-amplitude group, a constant-duration group, and a constant-average-velocity group). We hypothesized that average velocity would influence the location of the psychophysical curve relating acceleration judgments to the velocity ratio, which would mean that average velocity biases perception of the velocity ratio and that the velocity ratio does not represent the unique perceptual variable used by observers to perceive acceleration.

### Method

#### Participants

A group of 30 volunteers (19 men and 11 women) between 18 and 30 years of age (*M* = 21.7 years, *SD* = 3.2) participated in the experiment. All of the participants had normal or corrected-to-normal vision and gave informed consent before starting the experiment. Ten participants each were assigned to a constant-amplitude group, a constant-duration group, and a constant-average-velocity group, respectively.

#### Apparatus

Animations of accelerating or decelerating dots were presented on a 22-in. CRT monitor (Iiyama Vision Master Pro 514 HM204DT) at a 60-Hz frame rate. The experiment was programmed in MATLAB 5.2 using the Psychophysics Toolbox (Brainard, 1997; Pelli, 1997) running on an Apple Powerbook. Among other things, the Psychophysics Toolbox was important for controlling the timing of the animations; it enabled us to make sure that the delivery of each frame was synchronized with a refresh of the monitor screen.

**Fig. 3:**
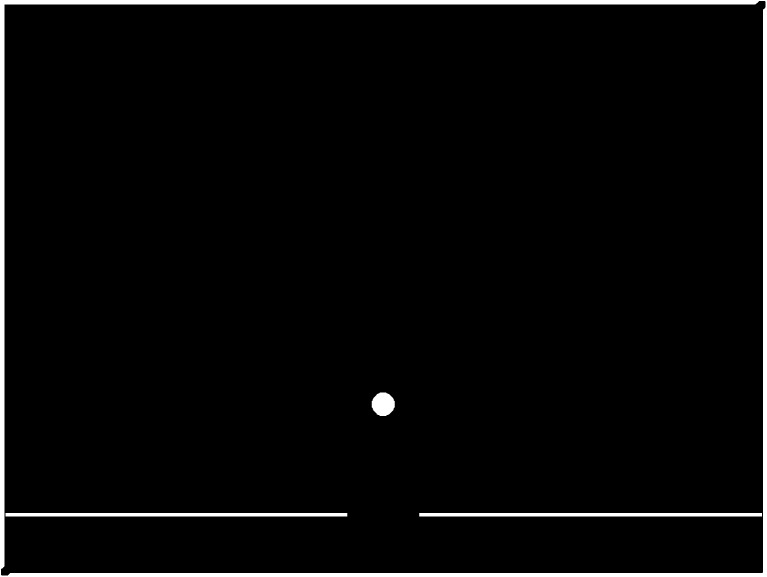
The display that was shown to the observers. The white dot started between the two horizontal lines at the bottom of the display and moved upward

The animations consisted of white dots moving up the screen across a black background (Fig. 3). Each frame of the animation was drawn in a 600 × 800 pixel area, which completely filled the monitor screen (100 pixels equaled 4.87 cm). A white horizon line was partially drawn at 60 pixels from the bottom of the frame. The diameter of the moving white dot was 20 pixels, and the dot kept that size during the entire animation.

We asked participants to maintain their chin in a chinrest to keep the distance from the point of observation to the monitor constant at 45 cm. The eyes of the participants were positioned at the height of the horizon line at the monitor screen.

#### Design

At the start of each trial, the white dot appeared at its initial position at the height of the horizon line and stayed there for 1 s before it started to move upward with a constantly decreasing or constantly increasing velocity. The velocity profiles were chosen such that the velocity ratios were –0.7, –0.5, –0.3, –0.1, +0.1, +0.3, +0.5, and +0.7 (negative values indicate decreasing velocity, and thus deceleration, and positive values indicate increasing velocity, and thus acceleration).

Apart from the velocity ratios, three aspects of the animations were varied across conditions: movement amplitude, movement duration, and average velocity. When the rate of change of velocity is kept constant, as we did in this experiment, keeping constant one of these variables means covarying of the two other variables. We had three groups of participants. A *constant-amplitude* group watched animations in which average velocity was manipulated by varying movement duration while keeping movement amplitude constant; a *constant-duration* group watched animations in which average velocity was manipulated by varying movement amplitude while keeping movement duration constant; and a *constant-average-velocity* group watched animations in which stimulus duration and movement amplitude covaried such that average velocity was constant across conditions. Table 1 gives the different combinations of movement amplitudes, movement durations, and average velocities presented to the three groups in the experiment.

**Table 1:** Durations, amplitudes, and average velocities of the stimuli presented to the observers of the three groups in our experiment

Stimulus Set	Duration (s)	Amplitude (px)	Average Velocity (px/s)
Constant-Amplitude Group			
S1	0.75	480	640
S2	1.00	480	480
S3	1.25	480	384
Constant-Duration Group			
S1	1.25	480	384
S2	1.25	384	307
S3	1.25	288	230
Constant-Average-Velocity Group			
S1	0.75	288	384
S2	1.00	384	384
S3	1.25	480	384

Eight levels of velocity ratio were factorially combined with three levels of movement amplitude, movement duration, or average velocity (depending on the specific conditions in each group), resulting in 24 different stimuli per group. With the order of presentation of the 24 conditions randomized within a block, the participants performed 12 blocks for a total of 288 trials. The first two blocks were considered familiarization blocks and were not used for the analyses.

#### Procedure

Each trial consisted of the presentation of a stimulus, followed by the presentation of a response window. We asked the observers to check one of two radio buttons, indicating whether they had seen an *increase* in velocity (acceleration) or a *decrease* in velocity (deceleration). After being satisfied with their choice, they were to click a “done” button, after which the next trial started.

#### Analysis

For each level of velocity ratio and each level of the manipulated stimulus variable (movement amplitude, movement duration, or average velocity, depending on the specific conditions in each group), we determined the number of “acceleration” judgments as a percentage of the number of repetitions of each trial. The effects of the manipulations were determined by entering these percentages, after arcsine transformation^6^ to meet the requirements of normality, into a repeated measures analysis of variance (ANOVA) with the factors Velocity Ratio (eight levels) and Manipulation (three levels) for each group. We will use generalized eta-squared (*η*_G_^2^) values to report effect sizes (Bakeman, 2005); *η*_G_^2^s of .02, .13, and .26 indicate small, medium, and large effects, respectively (cf. Cohen, 1988). Because the effects of velocity ratio seemed obvious and because we were interested in the effects of our various manipulations, we will not separately report the main effects of velocity ratio (all *F*s > 20, *p*s < .001, *η*_G_^2^s > .5), but restrict ourselves to the main effects of manipulation and the Velocity Ratio × Manipulation interaction effects. When Mauchly’s test indicated violations of the assumption of sphericity, we will report *F* values with degrees of freedom adjusted using Greenhouse–Geisser epsilons.

### Results

**Fig. 4:**
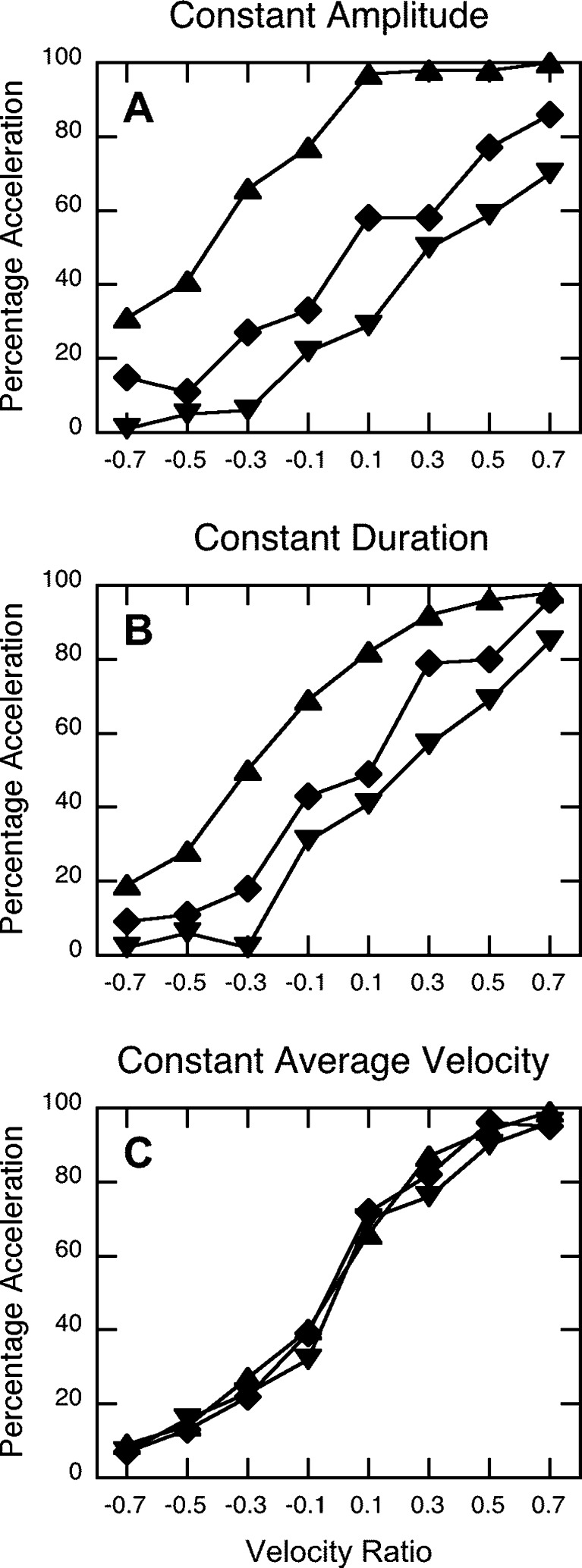
Percentages of acceleration judgments, averaged across observers, as a function of velocity ratio for the different groups and different values of stimulus duration, stimulus amplitude, and average velocity in the experiment. See Table 1 for all of the details regarding these three variables in the different conditions: , stimulus set S1; , stimulus set S2; and , stimulus set S3

Figure 4A presents the average percentages of acceleration judgments of the constant-amplitude group for each of the three levels of paired movement duration and average velocity, as a function of the eight levels of velocity ratio (negative velocity ratios indicate deceleration and positive velocity ratios indicate acceleration). As can be seen in Fig. 4A, the psychometric curves for the three different movement amplitudes and average velocities were clearly different. The ANOVA demonstrated that these differences were statistically significant: We found a large manipulation effect, *F*(1.07, 9.59) = 24.36, *p* < .005, *η*_G_^2^ = .47, as well as a small to medium Manipulation × Velocity Ratio interaction, *F*(14, 126) = 3.46, *p* < .05, *η*_G_^2^ = .06.

Figure 4B presents similar results for the constant-duration group, which had watched animations in which average velocity had been manipulated by varying movement amplitude. Again, the psychometric curves for the three pairs of average velocity and movement amplitude did not overlap. The ANOVA revealed a large manipulation effect, *F*(1.07, 9.60) = 20.17, *p* < .005, *η*_G_^2^ = .35, and a significant Manipulation × Velocity Ratio interaction, *F*(14, 126) = 2.25, *p* < .01, *η*_G_^2^ = .06.

Thus, when average velocity differed across conditions, either by varying movement duration or by varying movement amplitude, the psychometric curves in Fig. 4 were different. However, when movement duration and movement amplitude covaried in such a way that average velocity was constant across conditions, the curves overlapped (Fig. 4C); in this case, the ANOVA did not reveal any significant effect involving the factor Manipulation (*p*s > .25). Thus, the differences seen in the top two panels of Fig. 4 were most likely caused by the differences in average velocity and not by the differences in movement amplitude or movement duration in their own rights.

### Discussion

The first goal of the present experiment was to replicate Babler and Dannemiller’s (1993) somewhat underreported result that the average velocity of presented stimuli had an effect on the locations of psychometric curves of the percentages of judged acceleration as a function of the velocity ratio of the stimuli. Our results corroborate the findings reported by these authors. As did Babler and Dannemiller in their third experiment, we varied average velocity by varying both movement amplitude and movement duration. In addition, we also included a condition in which movement amplitude and movement duration covaried in such a way that average velocity was constant across all stimuli. Whereas Babler and Dannemiller were not in a position to report whether their average-velocity effect could be attributed to their manipulation of either movement amplitude or movement duration, our experiment was designed to identify each factor’s contribution to the observed average-velocity effect. The analyses showed large manipulation effects for the constant-amplitude group as well as for the constant-duration group, but not for the constant-average-velocity group, implying that the factor that made the psychometric curves differ was differences in average velocity across the stimuli. As soon as we manipulated this variable, effects were found on the locations of the curves, whereas varying movement amplitude or movement duration had an effect only if it was accompanied by a manipulation of average velocity.

Higher average velocities went with curves positioned more to the left. That is to say, stimuli with higher average velocities were judged more often as having accelerating motion. Note that this led to judgment errors that systematically varied with the average velocities of the stimuli. Clearly, what the observers responded to was a combination of the velocity ratio and average velocity. In other words, the velocity ratio was not the unique variable that determined the acceleration judgments of the observers. A search for a single variable that would predict the acceleration judgments of observers would combine the velocity ratio and average velocity (or their constituents). Whatever this variable may be, it seems clear that simply using sensitivity thresholds for the velocity ratio in the analyses presented by Brouwer et al. (2002) is problematic. Because the velocity ratio is not the single variable that observers respond to when judging acceleration and deceleration, we have to conclude that the Brouwer et al. (2002) study determined thresholds for the sensitivity of a variable that does not adequately capture the way that the visual system detects acceleration.

## Conclusion

In this contribution, we critically examined two assumptions made by Brouwer, Brenner, and Smeets (2002) in a study in which they concluded that optical acceleration is not the information used to control running to catch fly balls. Brouwer et al. (2002) first determined a perceptual threshold for human observers’ sensitivity to acceleration and then showed that the levels of acceleration when catchers started moving in the right direction were generally below the acceleration thresholds that had been established. The implication was that the movement initiation observed could not have been based on the use of the acceleration variable for which sensitivity thresholds had been determined.

The first assumption of the Brouwer et al. (2002) analysis that we investigated was the assumption of negligible air friction. We examined the effect of this assumption by comparing the optics associated with parabolic flight trajectories with the optics of more realistic flight trajectories resulting from the presence of air resistance. The results of this analysis revealed that the parabolic-flight-trajectory assumption biased Brouwer et al.’s (2002) analysis toward a rejection of optical acceleration as the information for knowing in which direction to start running. Adopting the more realistic assumption of balls experiencing air friction considerably weakened the argument of Brouwer et al. (2002).

The second assumption examined was the use of the velocity ratio (i.e., the change in velocity over the average velocity) as the perceptual variable used to detect acceleration. We experimentally demonstrated that, when asked to indicate whether a moving dot increases or decreases its velocity (i.e., accelerates or decelerates), observers do not base their judgments on this velocity ratio: In line with Babler and Dannemiller (1993, Exp. 3), we found that average velocity influenced the location of the psychophysical curve relating acceleration judgments to the velocity ratio. Whether one concludes from these findings that average velocity biases perception of the velocity ratio or, as we contend, that the velocity ratio does not represent the perceptual variable used by observers to perceive acceleration is inconsequential for the conclusion that Brouwer et al.’s (2002) use of a threshold for the perception of acceleration based on the velocity ratio was therefore not appropriate. As a result of this inappropriate assumption, the study does not allow for any firm conclusions to be drawn with respect to the question of whether optical acceleration can be used to initiate and guide a catcher’s movement.

Other assumptions made by Brouwer et al. (2002) have been considered elsewhere. For example, the effect of head rotation on the sensitivity for optical acceleration, as suggested by Zaal and Michaels (2003), has been studied by Bongers and Michaels (2008; see also Brouwer et al., 2006). Bongers and Michaels found effects of restrictions of head and/or eye movements (by blocking these with a neck brace and slit goggles, respectively) on both accuracy and response times. Interestingly, the effects were different when comparing a judgment task with a real catching task (see also Bootsma, 1989; Michaels, 2000; Michaels, Withagen, Jacobs, Zaal, & Bongers, 2001; Zaal & Michaels, 2003). The implication is that Brouwer et al.’s (2002) use of acceleration threshold values for real catching might be inappropriate because those threshold values were determined in a judgment task. Finally, in their psychophysics experiments, Brouwer et al. (2002) assumed that constant velocity is seen when a dot moves along the computer screen with the same velocity throughout its course (i.e., with a constant velocity). Perhaps surprisingly, this assumption is not true either. As demonstrated by Runeson (1974, 1975), for a stimulus to be judged to move at constant velocity, the motion should start with an acceleration followed by a plateaued velocity. Whether this is true only for stimuli in psychophysics studies in which the constancy of velocity must be judged, or also applies more generally, is not known. It is likely, though, that this phenomenon would play a role in an experimental setting in which observers have to adapt the acceleration of a moving dot such that it appears to move at a constant velocity (as in Exp. 2 of Brouwer et al., 2002).

In sum, we examined some of the underlying assumptions of Brouwer et al.’s (2002) conclusion that the Chapman strategy, of canceling optical acceleration, cannot be the strategy used in running to catch fly balls. We demonstrated that the assumption of negligible air resistance biased this conclusion. We also showed that observers do not base their acceleration judgments on the velocity ratio. Because Brouwer et al. (2002) assumed that in real catching the decision to start running forward or backward is based on detecting acceleration through this velocity ratio, they computed threshold times based on this variable. We have argued that the use of these threshold times is problematic. All in all, we infer that the strong conclusion presented by Brouwer et al. (2002) was unfounded. We therefore see no reason at this point to abandon the Chapman strategy.

## Footnotes

1 For reasons of convenience, we will consider that the fielder is facing the direction from which the ball comes.

2 Note that what needs to be detected in the context of the Chapman strategy is whether or not optical velocity is constant. That is to say, the term *optical acceleration* refers to *any* change in optical velocity. In the remainder of this article, we will use the term *optical acceleration* in this sense, unless otherwise indicated.

3 Incidentally, Brouwer et al. (2002) determined the time to reach velocity-ratio thresholds of 0.25 by looking for when optical velocity had reached a value of either 1.29 or 0.78 times the initial optical velocity, for balls crossing eye level behind and in front of the stationary participant, respectively. This method amounts to assuming that optical velocity changes linearly over time. For instance, at the moment that optical velocity equals 1.29 times the initial optical velocity (*v*_0_), the increase in optical velocity (0.29*v*_0_) divided by the average optical velocity over the interval until this moment (1.145*v*_0_) results in a velocity ratio of 0.25. Although the assumption of linearly changing optical velocity does not hold for balls even if they did fly in parabolic trajectories (see Fig. 2B; cf. Babler & Dannemiller, 1993; Zaal & Michaels, 2003), the picture as presented in Fig. 1A would not be very different from the picture drawn with the method of computing correct velocity ratios as a function of time. Thus, to ensure that we replicated Brouwer et al.’s (2002) result, Fig. 1A was created using Brouwer et al.’s (2002) method.

4 In our discussion of previous work, we will restrict ourselves to studies with stimuli that were moving visual objects (e.g., dots) that experienced a continuous change in velocity (i.e., we will not discuss studies that, for instance, have used stepwise changes in velocity or gratings rather than moving visual objects).

5 Previous studies on the perception of acceleration have also used variables by the name of *velocity ratio*. Schmerler (1976) defined a velocity ratio that was the ratio of the final velocity of the stimulus and the initial velocity. Hick (1950) and others after him (e.g., Filion, 1964; Notterman et al., 1971; Notterman & Page, 1957) used a velocity ratio that was the change in velocity divided by the initial velocity. In the case of linear changes in the velocity of the stimulus, all three versions of the velocity ratio are related. That is to say, given a constant rate of change of velocity, substituting any of the other two versions for Babler and Dannemiller’s (1993) version of the velocity ratio would also yield overlapping curves.

6 The figures report actual percentages rather than the arcsine-transformed values.
